# Persistent Upbeat Positional Nystagmus in a Patient with Bilateral Posterior Canal Benign Paroxysmal Positional Vertigo

**DOI:** 10.1155/2019/4281641

**Published:** 2019-03-26

**Authors:** Akihide Ichimura, Shigeto Itani

**Affiliations:** ^1^Department of Otorhinolaryngology, Tokyo Medical University, 6-7-1 Nishishinjuku, Shinjuku-ku, Tokyo 160-0023, Japan; ^2^Ichimura ENT Clinic, 2-11-10 Nishiwaseda, Shinjyuku-ku, Tokyo 169-0051, Japan

## Abstract

Here, we report a patient with persistent positional upbeat nystagmus in a straight supine position with no evident abnormal central nervous system findings. A 43-year-old woman with rotatory positional vertigo and nausea visited our clinic 7 days after the onset. Initially, we observed persistent upbeat nystagmus in straight supine position with a latency of 2 s during the supine head roll test. However, an upbeat nystagmus disappeared on turning from straight to the left ear-down supine position, and while turning from the left to right ear-down position, an induced slight torsional nystagmus towards the right for >22 s was observed. In the Dix–Hallpike test, the left head-hanging position provoked torsional nystagmus towards the right for 50 s. In prone seated position, downbeat nystagmus with torsional component towards the left was observed for 45 s. Neurological examination and brain computed tomography revealed no abnormal findings. We speculated that persistent positional upbeat nystagmus in this patient was the result of canalolithiasis of benign paroxysmal positional vertigo of bilateral posterior semicircular canals.

## 1. Introduction

Upbeat nystagmus is a sign of a central nervous system (CNS) disorder [1]. However, positional upbeat nystagmus can rarely be caused by peripheral lesions, such as benign paroxysmal positional vertigo (BPPV) of bilateral posterior semicircular canals [2, 3]. In patients with BPPV of bilateral posterior semicircular canals, positional upbeat nystagmus is typically observed as a transient positional nystagmus characterized by latency and habituation [2]. We report a patient with no CNS abnormalities who exhibited persistent positional upbeat nystagmus in a straight supine position during the head roll test with no spontaneous or gaze-evoked upbeat nystagmus. We speculated that the persistent positional upbeat nystagmus was caused by canalolithiasis of BPPV of bilateral posterior semicircular canals.

## 2. Case Presentation

**Figure 1:**
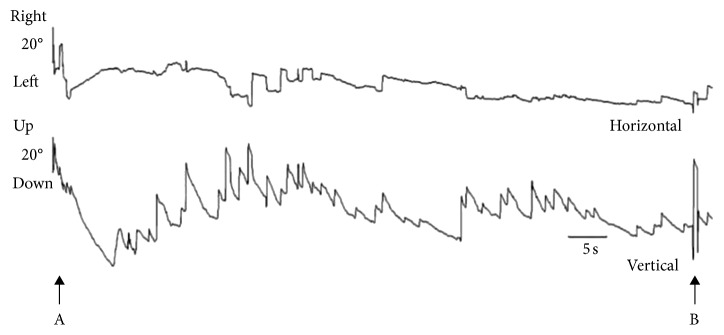
Video-oculographic recording of upbeat nystagmus (maximum slow phase velocity about 9.0°/s) in a straight supine position during the head roll test. A: the moment of the straight supine position and B: the moment of turning the head position from straight to the left ear-down supine position.

A 43-year-old woman complained of rotatory positional vertigo and nausea in the morning, particularly when lying down. She was examined at a local emergency department, on the same morning. Neurological examination and brain computed tomography (CT) revealed no abnormal findings, and she was discharged. Owing to persistence of symptoms, she visited our clinic 7 days after the onset. She denied any history of hearing loss, tinnitus, headache, or facial neurological symptoms. She had a history of BPPV 3 years ago. Her past medical, surgical, and family history was unremarkable; there was no history of head trauma. On examination, there was no dysdiadochokinesis, dysmetria, or tremors. Her gait was not ataxic, and there was no spontaneous or gaze-evoked nystagmus. Pure tone audiogram, neurological, and eye movement examinations, including tests of eye tracking, saccades, and drum optokinetic nystagmus test, were normal. Otolithic function was tested using cervical vestibular evoked myogenic potentials (VEMPs); ocular VEMPs showed no pathological findings. The positional and positioning nystagmus test, including the supine head roll and the bilateral Dix–Hallpike tests, was recorded using an infrared charge-coupled device camera. The supine head roll test revealed upbeat nystagmus for >110 s with a latency of 2 s on changing from the upright seated to straight supine position (Figure 1). Video-oculography was performed using the public domain software ImageJ and a Windows computer [4]. Head position was changed from the upright seated to the straight supine position by tilting the backrest of the electric chair backwards over a period of 8 s. In the supine head roll test, turning from the straight to the left ear-down supine position led to immediate disappearance of upbeat nystagmus; on turning from the left to the right ear-down position, slight torsional nystagmus towards the right was observed for >22 s with a latency of 6 s. In the Dix–Hallpike test, left head-hanging position provoked torsional nystagmus towards the right for 50 s with no latency. In the prone seated position, downbeat nystagmus with the torsional component towards the left was observed for 45 s with a latency of 3 s. The right head-hanging and upright seated position during the Dix–Hallpike test did not provoke nystagmus. Ten days after the onset, nystagmus and vertigo disappeared with no medical or physical treatment.

## 3. Discussion

We speculate that canalolithiasis of bilateral posterior semicircular canals was the pathophysiological basis of persistent positional upbeat nystagmus in this patient. This case report provides two clinical suggestions. Firstly, persistent positional upbeat nystagmus can be caused by peripheral lesions. Secondly, canalolithiasis of BPPV of the posterior semicircular canal may cause nystagmus lasting >60 s.

We speculate that positional upbeat nystagmus during the supine head roll test was caused by stimulation of the bilateral posterior semicircular canals due to change from the upright seated to the straight supine position. During the Dix–Hallpike test, torsional nystagmus towards the left in the left head-hanging position suggests stimulation of the left posterior semicircular canal, i.e., BPPV of the left posterior semicircular canal. In addition, torsional nystagmus towards the right in the right ear-down supine position suggests stimulation of the right posterior semicircular canal; downbeat nystagmus with the torsional component towards the left in the prone seated position suggests inhibition of the right posterior semicircular canal, i.e., BPPV of the right posterior semicircular canal. Equivalent stimulation of bilateral posterior semicircular canals provokes upbeat nystagmus according to Ewald's first and third laws. We further speculate that BPPV of bilateral posterior semicircular canals was not caused by cupulolithiasis but by canalolithiasis. In the case of cupulolithiasis, persistent torsional nystagmus towards the affected ear without latency should be observed during the affected ear-down and straight supine positions [5]. However, in our patient, upbeat nystagmus was observed after latency of 2 s despite the slow change in head position from the upright seated to the straight supine position over a period of 8 s; in addition, it disappeared immediately on turning from the straight to the left ear-down supine position during the supine head roll test, which indicates that cupulolithiasis was not the cause of upbeat nystagmus. Provoked nystagmus due to canalolithiasis in case of BPPV of the posterior semicircular canal was reported to disappear in <60 s [6]; however, we speculated that the upbeat nystagmus may last for >60 s even in case of canalolithiasis because of the slow movement of otoliths in the semicircular duct owing to their large mass and due to partial narrowing of the bilateral posterior semicircular ducts. The upbeat nystagmus disappeared immediately while the head position was turned from the straight to the left ear-down supine position during the supine head roll test; this was likely attributable to the temporary stoppage of otoliths due to their jamming in the semicircular duct [7]. This mechanism also explains why nystagmus was not observed in the right head-hanging and upright seated positions during the Dix–Hallpike test.

Upbeat nystagmus is an indicative of bilateral lesions of upward vestibulo-ocular reflex pathway or bilateral lesions of perihypoglossal nuclei; these may be caused by multiple sclerosis, brainstem tumors, brainstem infarction, or Wernicke's encephalopathy [1]. However, neurological examination and brain CT findings were normal in our patient. Nystagmus and rotatory vertigo disappeared 10 days after the onset with no medical or physical treatment. Thus, we speculate that the nystagmus was not caused by CNS disorders, despite the fact that brain CT could not detect abnormalities in the posterior fossa.

In conclusion, we believe that, in our patient, canalolithiasis of BPPV of bilateral posterior semicircular canals resulted in persistent positional upbeat nystagmus. This case report illustrated that persistent positional upbeat nystagmus could potentially be caused by peripheral vestibular disorders in addition to CNS disorders. In addition, the duration of nystagmus can be >60 s not only in cupulolithiasis but also in canalolithiasis in unusual circumstances.

## Supplementary Materials

Supplementary Materials Video: the supine head roll test and the Dix–Hallpike test.
